# Certain Manifestations of Syphilis of Importance in the Practice of Dentistry

**Published:** 1896-11

**Authors:** Charles M. Whitney

**Affiliations:** Boston, Mass.


### International Dental Journal.

Vol. XVII. November, 1896. No. 11.

### Original Communications.[1]

### CERTAIN MANIFESTATIONS OF SYPHILIS OF IMPORTANCE IN THE PRACTICE OF DENTISTRY.[2]

BY CHARLES M. WHITNEY, M.D., BOSTON, MASS.[3]

In accepting the invitation to address this Society upon the subject of syphilis in its relation to the practice of dentistry, I found myself in the position of one who has no new or unfamiliar facts to present, but must content himself with a repetition of those already well known.

The importance of the subject, however, is so great that I was only too glad of an opportunity of refreshing your minds upon it, and of repeating certain facts which have been observed. Some of these I have already presented in a paper before another society of dentists, and from this I shall take the liberty of quoting at this time.

For more than four centuries there has been in existence in the world a destructive and dangerous disease, characterized by appearances more varied, and results more disastrous than almost any other with which we are acquainted.

---

[1] The editor and publishers are not responsible for the views of authors of papers published in this department, nor for any claim to novelty, or otherwise, that may be made by them. No papers will be received for this department that have appeared in any other journal published in the country.

[2] Read before the Massachusetts Dental Society, June 4, 1896.

[3] Surgeon to the Genito-Urinary Department of the Boston Dispensary.

Its very name, in its mythical origin, implies the lowest associations,—a companion of swine,—and its mere mention seems to suggest immorality and uncleanness. While in most instances there is a measure of truth in this view, it is not always the correct one, for the disease may, and has been, innocently acquired, and it is to this particular portion of the subject that I wish to especially call your attention.

In order that you may understand by what means this innocent transmission takes place it is necessary to call your attention to certain points in the natural history of the disease as it ordinarily manifests itself, and to consider by what means this danger can be avoided. Syphilis, by one not familiar with its wonderfully varied appearance, is frequently pictured as a disease accompanied by loathsome eruptions, by facial or bodily deformities so distinct that any one could recognize them, and therefore could avoid any danger of contamination for this reason alone; by a condition, in short, like that of leprosy, both repulsive and hideous. If such were the fact, it would add much to the safety of the community but it is not, for, in many instances the disease, from its onset to its conclusion, may be so mild that any one but a trained observer may overlook some of its most apparent symptoms. Syphilis, however, in spite of the variety of symptoms in its later stages, has at its onset tolerably well-defined laws of development.

It is a disease requiring for its origin inoculation of the virus from some pre-existing case upon a denuded surface, either epithelial or mucous. Wherever this inoculation occurs an absorption of the poison results by means of the lymphatic vessels, and the beginning of the first stage of the disease takes place. This primary inoculation does not require a large denuded surface; a minute opening no larger than a pin-point serves quite as well as a larger one, and it may be quite invisible to close inspection, and much more dangerous because so easily overlooked.

Inoculation, then, having occurred, one of two things happens; the point of entrance of the poison may heal, to reappear later, or it may slowly increase in size. In either case we have at the end of a period of about three weeks a local ulcer of varying size and appearance, the initial lesion of syphilis, commonly called the chancre. This apparently local sore exhibits wide variation in appearance, depending partly upon the size of the original abrasion and partly also upon the locality affected. Upon the finger a common situation is at the base of the nail, and there may be a reddened, swollen ulcer, with a slight watery discharge, upon the lip; however, where the tissues are soft, a thickening or induration occurs about the primary lesion, so that we have a hard, usually painless tumor, varying in size from a pea to a large cherry. When present, this induration, which is due to a development of granulation tissue, is extremely characteristic of syphilis, and upon mucous surfaces is rarely absent. As the lymphatic vessels slowly carry away the poison, it is stored up, we may say, in the glands nearest, and these enlarge. Thus, in the case of infection upon the lip, we look for an enlargement of the submaxillary gland upon the affected side. In infection upon the finger, a small gland above the elbow is enlarged.

Introduction of the specific poison into the system through a denuded surface, a period of delay or incubation, and the appearance of a local sore with enlarged glands in the vicinity mark, then, the first or initial stage of syphilis.

However obscure or slight the initial lesion may be, it is always present, for by this and by no other means—save in infants by heredity—can the disease be acquired. Not rarely the nature of these extragenital lesions is not recognized; the later manifestations are mistaken for other skin-diseases, and, being untreated, in later years we have the long train of suffering and deformity consequent upon untreated syphilis. Counting, then, three weeks for the first period of incubation, we come to a second and longer period of delay before further constitutional symptoms make their appearance. This usually occupies six weeks, during which the morbid process is apparently at a stand-still save for a slight increase in local symptoms and greater adjacent glandular enlargement. At the expiration of this period we have other symptoms, constitutional in character, which usher in the second stage of the disease. The first of these to appear is an eruption upon the skin, consisting of rose-colored spots, scattered thickly over the body, resembling somewhat the eruption of measles. At about the same time the throat becomes sore, and presents a reddened area, usually limited to the fauces, or extending forward to the junction of the hard and soft palate, where it is sharply limited. Later, there appear the mucous patches, which constitute the first and most important symptom of syphilis in its relation to the practice of dentistry, and to which I shall presently call your attention in more detail. With these symptoms we usually find more or less headache, occasionally feverish sensations, and feelings of general discomfort. The glands of the body generally enlarge, the hair upon the scalp and eyebrows may loosen and fall, and the original eruption may become scaly, or otherwise altered in character.

Under appropriate treatment these symptoms disappear, the skin-eruption gradually fades, leaving little or no trace of its presence, and the second stage of the disease is passed.

From this time the disease manifests no regularity of course, and save for an occasional mucous patch in the mouth there may be nothing to mark its presence in the system. Sooner or later, however, in untreated cases, the symptoms of the tertiary stage appear, and here for the first time we find evidence of the destructive and terrible nature of the disease. The limits of this paper will not allow me to more than briefly touch upon these, but it will suffice to say that no organ or structure is safe from its ravages. Its possibilities untreated are appalling.

Upon the skin may appear disfiguring and repulsive eruptions; ulcers of varying size and in varying locations, resulting in deformity and scars; the organs of special sense may be attacked, resulting in loss of sight or hearing; by disease of the throat the voice may be impaired or lost, and a hoarse whisper be all that is left of a clear voice; the bones become inflamed, resulting in necrosis, which may be present in the nasal bones, causing frightful deformity by loss of the whole nose; the brain may be involved, resulting in loss of reason or paralysis; tormenting neuralgias, local and general, mark its effect upon the peripheral nerves. All these and many more make up the list of the dreadful possibilities of the disease when neglected and allowed to pursue its own ways. The most strange and unfortunate thing about this disease is its latency, there being no way of determining when an outbreak may occur, for after many years of fancied security, any of these dreadful calamities may result. Preventable, if their character is discovered in season, they are often beyond the help of the physician in saving the disastrous effect of their presence in important organs or structures.

Admitting, then, that these are the possibilities of syphilis, what especial interest have they for the dentist, whose professional attention is directed to the mouth, and not to the eyes or throat, to the skin or bones, to the brain or other visceral organs?

A most important relation indeed, for it is the mouth lesions of syphilis which are the most potent agents in its innocent transmission,—by far the most important because the least understood, and therefore not guarded against. Until within a few years this subject has received but little attention. Occasional cases have been reported of mouth infection, and every physician has been aware of these dangers; but the patient, but little instructed regarding its precise nature, has inoculated innocent persons, and these again others, and thus the vicious circle has widened.

What form, then, does syphilis take in the mouth that we should fear it so greatly, and that contamination should so frequently result?

At the time of the secondary symptoms there appear various changes in the mouth and throat, to only one of which I wish especially to call your attention,—the mucous patch, as it is termed. Analogous to the rose-spots upon the skin, modified only by the different structure of the lining of the mouth, there appear white spots of various sizes, situated upon the inner surface of the lips, especially where they are brought in contact with an irritating point like a decayed tooth, along the sides of the tongue, upon the tonsils and roof of the mouth. Making their first appearance then, they are the most frequent of any of the symptoms of the disease, and constantly reappear during its course whether treated or not. Their size is variable, and they are only moderately sensitive. They are covered with a whitish, opaline film, which gives them the appearance of having been touched with caustic.

They are not unlike the frequent and harmless "canker spots," and very often cannot be distinguished from them. The chief points of difference are that the canker spots are more of an inflammatory type, yellowish spots with a reddish circumference, and they are much more sensitive.

Besides these most common and dangerous manifestations of syphilis in the mouth, we have other and later symptoms. Deep ulcerations may occur upon the mucous membranes, with destruction of tissue and deep scars. Gummata, which occur as a late symptom in various parts of the body, occur in the mouth and throat, and consist in one or two small, hard tumors, which very rapidly degenerate, leaving an open ulcer, and when they occur upon the palate or roof of the mouth very frequently perforate the tissues separating the mouth and nose, and a very troublesome condition results, requiring the aid of the dentist in many instances. The early symptoms already described are the most dangerous to the dentist, for it must not be forgotten that not alone are the spots themselves contagious, but the saliva also, and may convey the poison to any part which it touches. How important, then, for the dentist to use proper care in these mouths, with his fingers constantly bathed in a solution of syphilis, if I may be allowed the phrase!

How shall the dentist, if a patient presents himself with spots in the mouth, distinguish the harmless from the dangerous form?

Bearing in mind the other symptoms most common, it is well to look for any swelling of the glands behind the ears, for loss of hair or eyebrows, for crusts upon the scalp, and scars of other ulcerations in the mouth, especially at the corners. These may be all absent, and I do not hesitate to say that, whenever any whitish spots are seen in the mouth of a patient, they should be viewed with suspicion, and proper measures for protection taken in every case, whether further evidences of syphilis be present or not. Furthermore, it seems to me to be the obvious duty of every physician having a case of syphilis under his care to advise the patient to refrain from having any dental work done until the contagious stage is passed, or, at any rate, until all local signs have disappeared from the mouth. Remembering, then, that syphilis in a most contagious form may exist in the mouth, and show nowhere else evidence of its presence, we can readily understand that the disease may be innocently acquired or transmitted in two ways,—either by direct contact or by the deposition of the saliva or virus from any lesion upon any object which may be brought in contact with a denuded surface.

For a proper understanding of the subject the medical profession is greatly indebted to Dr. L. Duncan Bulkley, of New York, whose labors have been incessant in collecting all available evidence upon the subject, and the result of which he has compiled in a recent work, entitled "Syphilis Insontium, or Syphilis in the Innocent," and it is from this classic that I have obtained many of the facts which I shall present for your consideration. Instances of direct transmission are of frequent occurrence; kissing has been the cause of many chancres of the lips, and no one who has seen many cases of this disease has failed to observe illustrations of this form of infection. Tooth-wounds, contracted in various ways, as by blows or by biting, have been followed by local and general syphilis. I have personally observed two instances of this method. Sucking wounds and removing foreign bodies from the eyes with the tongue have produced the same untoward result.

In considering the possibility of infection by indirect means we cannot but be amazed at the variety of ways in which this is brought about. Any article upon which can be deposited the diseased virus can produce this effect, and we find instances in almost all occupations. The glass-blower, passing the tube from mouth to mouth; the musician, using the flute or cornet; the shoemaker, whose mouth is filled with pegs, to be thrown down and used by an uninfected individual; the artisan, with the blow-pipe, have all been the means of infecting their especial implements, through which others have become infected. The pencil, wet in the mouth of one infected, spreads the disease to the innocent.

There is, apparently, no limit to possibilities of these methods. Tooth-brushes, toothpicks, clothing, towels, pipes, razors, whistles, spoons, pins, coins, drinking vessels, in short, any of the articles used in industrial or domestic life have become infected, and have contributed to the number of cases of extragenital syphilis. In Finland two hundred cases were infected by the operation of wet-cupping, and hundreds of other cases have been ascribed to the same cause in other countries. Over seventy were infected in Paris by the Eustachian catheter of an otologist. A short time since a patient was referred to me, who had a typical chancre of the left nostril and well-marked eruption over the body; its origin was in doubt, but may have been due to the use of a spray-tube or contaminated towel. In estimating the importance of mouth lesions in the production of innocent syphilis, it is only necessary for me to call your attention to the statistics of Dr. Bulkley. He has collected nine thousand and fifty-eight cases of extragenital infection, and of these there were fifteen hundred and four in the mouth and throat, and of the remainder three thousand two hundred and forty-nine suggest from their location the possibility of this source of infection, making a total of four thousand seven hundred and fifty-three cases, or more than half of all the cases of this kind. When we consider that not one in fifty which occurs is reported in medical literature, we can form some idea of the probable number of cases. When it is remembered also that the innocent acquirement of syphilis is the exception and not the rule, we can form some estimate of the thousands of cases which have been in the past and are in the present.

From its nature, kept as secret as possible, it is impossible to estimate the prevalence of syphilis in the community, and it is to hospitals that we must look for some degree of definiteness upon the point. Even here, however, we have only the evidence of the special departments, and of such other cases as bear evidence of the disease when treated for other complaints, which are often not placed on record.

Dr. Armstrong, in Morrow's "System of Genito-Urinary Surgery, Syphilology, etc.," has investigated the subject, and his results are interesting.

In a New York hospital, St. Luke's, which serves as a type of hospitals everywhere, about one per cent. of cases treated were syphilitic, and of those refused admission about six per cent. were similarly affected. In the United States Marine Hospital service, where thirty to fifty thousand cases are annually treated, about ten per cent. were syphilitic. In the United States army, one and a half per cent., in the navy about two per cent. Accepting one to two per cent. as the mean, and applying it to a city of the size of Boston, where in the vicinity of one hundred thousand patients are annually treated in the various hospitals, we find from one to two thousand cases of syphilis prevalent in the community, and I do not think it any exaggeration to say that not one or two, but many thousand cases are present in this and every other large city.

Realizing that each case may inoculate many others, innocently or otherwise, we cannot ignore the danger because we do not see it or neglect precautions which may seem unnecessary.

Among the many instances of acquiring or transmitting innocent syphilis, how many have we which directly concern the profession which I have the pleasure of addressing to-day? How many dentists have been inoculated in operating upon syphilitic patients? Availing myself of Dr. Bulkley's work, I find there are not many instances on record, but even a single case is an extremely instructive object lesson. In the *Boston Medical and Surgical Journal* of some years ago, a dentist reports his own case. Infection occurred on the left hand, above the nail, in the middle finger.

Neumann reports a case from a wound on the finger, produced by the sharp edge of a tooth. Otis and Hutchinson report each a case, and Bumstead states that he knows of several cases.

It is not necessary to multiply cases to prove a proposition so simple, and the very nature of the disease renders the subject of it very loath to add to our knowledge by reporting his case.

Of instances of conveying the disease upon dental instruments we have some ten or twelve cases, nearly all being produced by extraction of teeth.

Admitting, then, these dangers, how shall they be avoided? By what means shall the dentist avoid inoculation himself, or prevent a possible infection of his patients? As regards the first, careful inspection of the fingers, especially about the roots of the nail, the site of the common "hang-nail," as it is termed. Every abrasion should be thoroughly brushed with contractile collodion. Should any escape observation in working upon such a case, the spot should be thoroughly washed with soap and water and touched with one-per-cent. solution of corrosive sublimate or pure carbolic acid.

As regards the second possibility, all instruments should be frequently and thoroughly cleansed, and after working upon such a case, should be sterilized by boiling water, to which has been added one-per-cent. washing soda (a tablespoonful to a pint) to prevent rusting of the instruments. If I were to select any special instrument deserving care, I should select the mouth-mirror, which cannot be sterilized or rendered antiseptic by chemical means. In such cases an individual mirror should always be used, as has already been suggested by Dr. Henry L. Upham, a member of this Society.

The mouth-piece used in administering gas should be very carefully watched and kept absolutely clean. Instruments may be rendered fairly safe by immersion in two-per-cent. creolin solution, or pure carbolic acid. By such precaution much of the danger to the dentist can be averted, and so unfortunate a circumstance as infecting a patient can be avoided.

Having thus briefly sketched some of the chief points in relation to syphilis, I desire to emphasize these points: that we have in the disease one that is more prevalent in the community among all classes than is generally admitted; that the symptoms in the mouth are the earliest and most constant, and exist where there are no other; that these lesions have been the means of inoculating many innocent individuals in a variety of ways, and, finally, that dentists have not only been themselves inoculated, but have infected patients by means of their instruments. Knowing these facts, can we doubt the wisdom of instructing dental students in relation to the subject in order that so deplorable an accident as syphilitic infection should be guarded against. It is not my purpose to exaggerate the frequency of this disease or the dangers arising from it in the practice of dentistry, but to present the foregoing facts and invite you to make your own conclusions from them.

Sooner or later public attention must be called to the fact that the acquirement of syphills is not necessarily a disgrace, and that morality is not a certain means of avoiding it. When every public drinking glass is a menace, when innocent children may be inoculated by toys passed from mouth to mouth by one in whom the disease is present, by parental guilt or ignorance; when so simple a thing as wetting the pencil in the mouth has resulted in the development of the disease, surely it is time to look the matter squarely in the face and to describe in plain language its nature and its dangers. Until this is done, innocent victims must be sacrificed upon the altar of ignorance.

The guilty must suffer the consequences of their own acts, but speed the time when they and every one in the community shall have full knowledge of this disease, as of other contagious diseases, and thus be saved from its direful results.

If I can hope that I have presented any facts in this paper which are worthy of your serious thought I shall be well satisfied at having accepted your courteous invitation to address you upon this subject.

